# Development of a Reverse Transcription Loop-Mediated Isothermal Amplification Method for the Rapid Detection of Subtype H7N9 Avian Influenza Virus

**DOI:** 10.1155/2014/525064

**Published:** 2014-02-06

**Authors:** Hongmei Bao, Yuhui Zhao, Yunhe Wang, Xiaolong Xu, Jianzhong Shi, Xianying Zeng, Xiurong Wang, Hualan Chen

**Affiliations:** Animal Influenza Laboratory of the Ministry of Agriculture, State Key Laboratory of Veterinary Biotechnology, Harbin Veterinary Research Institute, Chinese Academy of Agricultural Sciences, 427 Maduan Street, Harbin 150001, China

## Abstract

A novel influenza A (H7N9) virus has emerged in China. To rapidly detect this virus from clinical samples, we developed a reverse transcription loop-mediated isothermal amplification (RT-LAMP) method for the detection of the H7N9 virus. The minimum detection limit of the RT-LAMP assay was 0.01 PFU H7N9 virus, making this method 100-fold more sensitive to the detection of the H7N9 virus than conventional RT-PCR. The H7N9 virus RT-LAMP assays can efficiently detect different sources of H7N9 influenza virus RNA (from chickens, pigeons, the environment, and humans). No cross-reactive amplification with the RNA of other subtype influenza viruses or of other avian respiratory viruses was observed. The assays can effectively detect H7N9 influenza virus RNA in drinking water, soil, cloacal swab, and tracheal swab samples that were collected from live poultry markets, as well as human H7N9 virus, in less than 30 min. These results suggest that the H7N9 virus RT-LAMP assays were efficient, practical, and rapid diagnostic methods for the epidemiological surveillance and diagnosis of influenza A (H7N9) virus from different resource samples.

## 1. Introduction

A novel reassortant avian influenza A (H7N9) virus emerged and spread among humans in China in March 2013 [1]. As of August 11, 2013, there have been 135 laboratory-confirmed human cases in eleven provinces in China, and 44 of these cases resulted in death [2]. Novel H7N9 viruses were simultaneously prevalent in chickens, pigeons, and in the environment of the live poultry markets [3]. The H7N9 virus poses a serious threat to public health [4]. Therefore, the development of a simple and rapid diagnostic method, which can quickly diagnose and timely monitor the H7N9 influenza virus, is extremely important. An accurate diagnosis will allow the timely administration of antiviral therapy and may enable the quarantining of infected cases to prevent the further spread of the virus [5].

Currently, several laboratory techniques for the detection of the H7N9 virus include molecular diagnostics, virus isolation, typing by hemagglutination inhibition and neuraminidase assay, and serology [6–11]. However, the isolation and identification of viruses require extended periods, which range from days to weeks, and do not meet the time requirements that are necessary for the prevention of epidemics. In addition, the H7N9 influenza causes disease among humans and is a significant threat for public health [12]; consequently, the virus must be handled in biosafety level 3 facilities. Therefore, rapid, sensitive, and specific molecular diagnostics have played important roles in the rapid detection of H7N9 influenza viruses. A real-time reverse transcription (RT)-PCR (rRT-PCR) assay has been used to detect H7N9 influenza virus RNA [6]. The real-time RT-PCR is performed in a closed system to minimize contamination. However, rRT-PCR requires an expensive machine system, primers/probes with special modifications, and experienced laboratory workers.

The loop-mediated isothermal amplification (LAMP) assay is a rapid, accurate, and cost-effective diagnostic method that amplifies the target nucleic acid under isothermal conditions [13]. This method does not require an additional reverse transcription step; only a water bath or heating block is required to amplify large amounts of nucleic acids in 30–60 min [14]. In addition, nucleic acid electrophoresis is not required to assess the results, which can be determined by visualizing a white magnesium pyrophosphate precipitate (easier to be observed after the addition of a fluorescent dye) [15]. Therefore, LAMP is easy to introduce in the frontline or poorly equipped laboratories. In the previous study, we developed the RT-LAMP method for the rapid detection of influenza virus subtype H7 [16]. This RT-LAMP assay has the capacity to detect both high- and low-pathogenic H7 AIV strains. In the present study, we evaluated the ability of H7-RT-LAMP to detect a novel influenza A (H7N9) virus that was identified in humans and birds in China and developed a novel one-step RT-LAMP assay that was specific for the neuraminidase (NA) gene of the H7N9 influenza virus. The H7N9 influenza virus RT-LAMP assays may enable the diagnosis of pandemic H7N9 influenza virus infection faster and more easily from clinical samples of different species.

## 2. Materials and Methods

### 2.1. Virus Strains

Reference influenza virus subtype strains N1–N9, H7N9 influenza virus subtype (A/chicken/Shanghai/S1053/2013(H7N9), A/pigeon/Shanghai/S1069/2013(H7N9), A/environment/Shanghai/S1088/2013 (H7N9)), and A/Anhui/1/2013 (AH/1) [1, 3], which were used in the present studies, were maintained and provided by the Animal Influenza Laboratory of the Ministry of Agriculture (Harbin, China) (Table 1). The Newcastle disease virus (NDV), avian infectious bronchitis virus (IBV), and infectious laryngotracheitis virus (ILTV) were provided by the State Key Laboratory of Veterinary Biotechnology, Harbin Veterinary Research Institute (HVRI), Chinese Academy of Agricultural Sciences (CAAS).

### 2.2. RNA Extraction

Genomic viral RNA was extracted using an RNeasy Mini Kit (Qiagen, Valencia, CA) according to the manufacturer's protocol. RNA isolation from the highly pathogenic avian influenza virus and H7N9 influenza viruses was performed in a biosafety level 3 laboratory at the Harbin Veterinary Research Institute. The RNA concentration (ng/*μ*L) was measured with a NanoDrop ND-1000 apparatus (Thermo Scientific).

### 2.3. H7-RT-LAMP Assay

The H7-RT-LAMP was performed with primers (outer primers (H7-F3 and H7-B3), inner primers (H7-FIP and H7-BIP), and loop primers (H7-LF and H7-LB)) that were specific for the H7 AIV HA gene, as described previously [16].

### 2.4. H7N9 Influenza Virus NA-Specific RT-LAMP Assay

**Figure 1:**
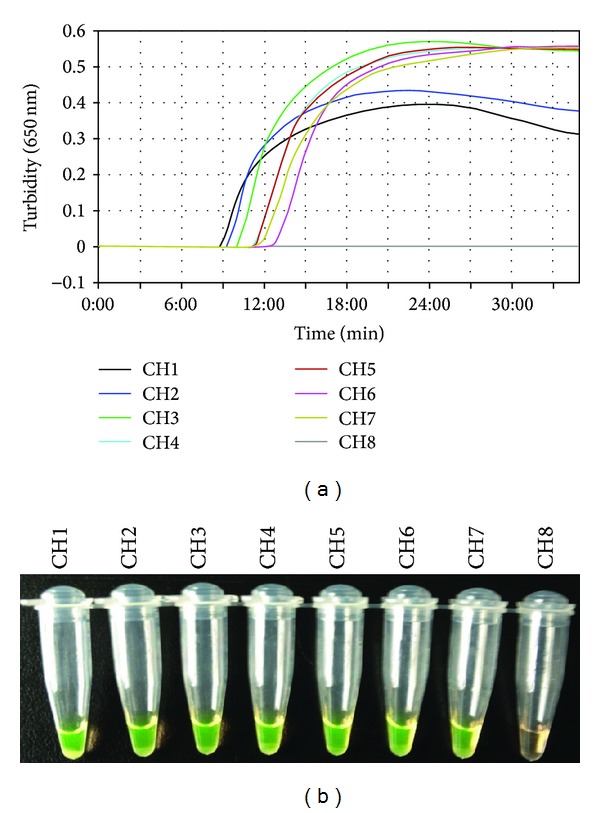
Detection of H7N9 influenza viruses by H7-RT-LAMP. Viral RNAs were extracted from the H7N9 influenza viruses and were amplified using H7-RT-LAMP. LAMP products were detected by a real-time turbidity assay using an LA-320c (a) and a fluorescence assay (b). CH1: A/CK/Shanghai/S1053/2013 (H7N9); CH2: A/EN/Shanghai/S1088/2013 (H7N9); CH3: A/PG/Shanghai/S1069/2013 (H7N9); CH4: A/DK/Anhui/SC702/2013 (H7N9); CH5: A/CK/Jiangsu/SC002/2013 (H7N9); CH6: A/Anhui/1/2013 (H7N9); CH7: A/African starling/England/983/79 (H7N1); CH8, negative control.

Primers that were specific for the NA gene of N9 influenza viruses were designed using the Primer Explorer version 4 software (Eiken Chemical Co., Ltd., Tokyo, Japan; http://primerexplorer.jp/elamp4.0.0/index.html) and synthesized by Shanghai Invitrogen Co., Ltd. A set of six primers included two outer primers (forward primer N9-F3 and reverse primer N9-B3), two inner primers (forward inner primer N9-FIP and reverse inner primer N9-BIP), and two loop primers (forward loop primer N9-LF and reverse loop primer N9-LB) (Figure 1). The RT-LAMP assay was performed in a final reaction volume of 25 *μ*L, which consisted of the N9-FIP and N9-BIP inner primers (1.6 *μ*M), as well as the N9-F3 and N9-B3 outer primers (0.2 *μ*M), as described previously (Bao). Amplification reactions were performed from 55°C to 65°C for 30 min and at 62.5°C for 30 min, 45 min, and 60 min using either an LA-320C Loopamp real-time turbidimeter (Teramecs, Japan) or a water bath. Respective mixtures were heated at 80°C for 10 min to terminate respective reactions. LAMP products were then evaluated by fluorescent detection with a fluorescent detection reagent (Eiken Chemical Co., Ltd.). A positive control (a sample known to be positive for the template) and a negative control (a sample devoid of template) were included in each reaction.

### 2.5. RT-PCR

RNA samples were amplified by RT-PCR using a one-step RNA PCR kit (Takara, Japan) with N9F and N9R primers (Table 2). The reaction conditions were set at 45°C for the reverse transcription for 45 min, 94°C for the predenaturation for 2 min, and then 35 cycles at 94°C for 30 s, 52°C for 45 s, and 68°C for 45 s, followed by the final extension at 68°C for 8 min. The RT-PCR products were subjected to electrophoresis on a 1.5% agarose gel, and the target bands were visualized by staining with ethidium bromide.

### 2.6. Sensitivity and Specificity of RT-LAMP Assay

The sensitivity of the RT-LAMP assay was tested with RNA that was extracted from serial 10-fold dilutions of H7N9 viruses. The species specificity of the H7N9 influenza virus NA-specific RT-LAMP assay was evaluated using RNA samples that were extracted from avian influenza reference N1-N9 subtype strains and other avian respiratory viral pathogens, including NDV, IBV, and ILTV. The H7N9 virus (A/chicken/Shanghai/S1053/2013(H7N9)) was used as a positive control.

### 2.7. Detection of Clinical Specimens

In total, 269 samples (including tracheal swabs and cloacal swabs) were collected in the experiment. Among these samples, 150 samples from chickens, 35 samples from pigeons, 30 samples from ducks, and 54 samples from the environment were collected from three live poultry markets in Shanghai in March, 2013, when the H7N9 influenza outbreak occurred there [3]. Each sample was placed in 2 mL minimal essential medium, which was supplemented with penicillin (2000 Units/mL) and streptomycin (2000 Units/mL). All samples were investigated using viral isolation, RT-PCR, and RT-LAMP. The virus isolation was performed in 10-day-old specific-pathogen-free embryonated chicken eggs [3]. All virus isolation procedures and RNA extractions were conducted in a biosafety level 3 facility, which was approved by the Ministry of Agriculture, China.

## 3. Results

### 3.1. Detection of the H7N9 Virus with the H7-RT-LAMP Assay

**Figure 2:**
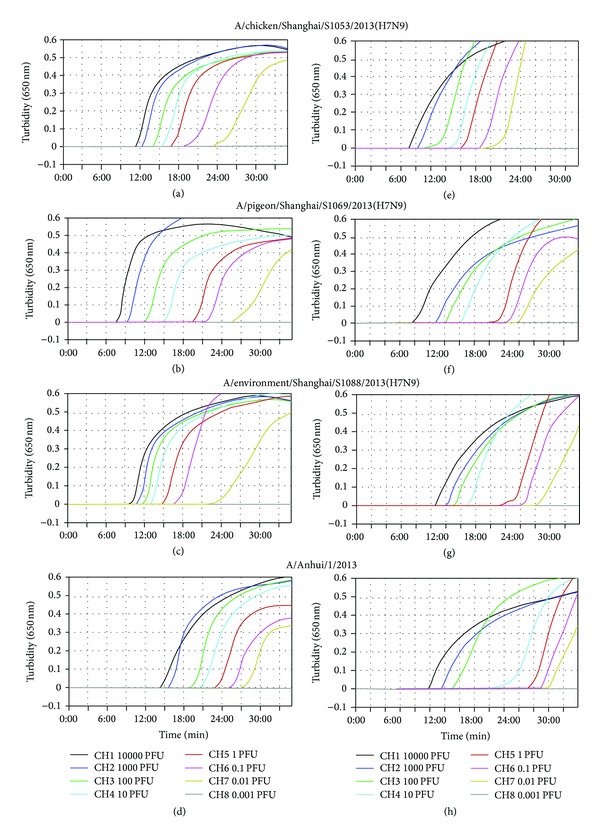
Sensitivity of H7-RT-LAMP and N9-RT-LAMP for the detection of H7N9 influenza viruses that were isolated from various sources. ((a) to (d)) A/chicken/Shanghai/S1053/2013 (H7N9), A/pigeon/Shanghai/S1069/2013(H7N9), A/environment/Shanghai/S1088/2013 (H7N9), and A/Anhui/1/2013 (H7N9) RNAs were amplified using H7-RT-LAMP. ((e) to (h)) A/chicken/Shanghai/S1053/2013 (H7N9), A/pigeon/Shanghai/S1069/2013 (H7N9), A/environment/Shanghai/S1088/2013 (H7N9), and A/Anhui/1/2013 (H7N9) RNAs were amplified using N9-RT-LAMP. Viral RNA concentrations ranging from 10,000 to 0.001 PFU per tube were tested. LAMP products were detected by a real-time turbidity assay using an LA-320c.

The H7-RT-LAMP assay was previously developed in our lab [16]. To evaluate the ability of the H7-RT-LAMP assay to detect a novel influenza A (H7N9) virus identified in humans and birds in China, H7N9 viruses that were isolated from chickens, pigeons, the environment, and humans were tested by the H7-RT-LAMP assay. All H7N9 influenza viruses can be detected in less than 30 min by this assay (Figure 1). The minimum detection limit of the H7-RT-LAMP assay was 0.01 PFU per reaction for the H7N9 influenza virus that was isolated from various sources (Figures 2(a)–2(d)). These results indicated that the H7-RT-LAMP assay could be used for the detection of H7N9 influenza viruses. Because the H7-RT-LAMP assay targets only the HA gene, this RT-LAMP method cannot identify the influenza virus NA subtype. To overcome this limitation, the H7N9 influenza virus NA-specific RT-LAMP (N9-RT-LAMP) assay was developed in this study.

### 3.2. Design of the N9-RT-LAMP Assay

The H7N9 influenza virus NA-specific RT-LAMP assay primers were designed from the alignments of the conserved sequence of the NA genes of 79 H7N9 influenza viruses (41 NA genes sequenced by our lab, 38 NA genes from GenBank), which were identified from chickens, pigeons, the environment, and humans [1, 3]. Conserved fragments with the highest levels of homology were chosen as templates for the design of H7N9 influenza virus RT-LAMP primers. Among these 79 NA gene sequences of H7N9 influenza viruses, 3 NA gene sequences had 1 mismatched nucleotide sequence in the N9-B3 primer and 1 NA gene sequence had 1 mismatched nucleotide sequence in the N9-RT-LAMP primer. The other H7N9 influenza virus NA gene sequences matched the NA-specific RT-LAMP assay primer sequences 100%. An RT-LAMP assay with the N9-specific primers was successfully developed and optimized. The optimum volumes of components in the reaction mixture included 1.6 mM each of FIP and BIP primers, 0.2 mM each of F3 and B3 primers, 1.2 mM dNTPs, 8 mM MgSO_4_, 0.2 M betaine, 8 U Bst DNA polymerase large fragment, and 1 *μ*L target DNA. The optimal temperature and time were 62.5°C for 30 min.

### 3.3. N9-RT-LAMP Sensitivity

**Figure 3:**
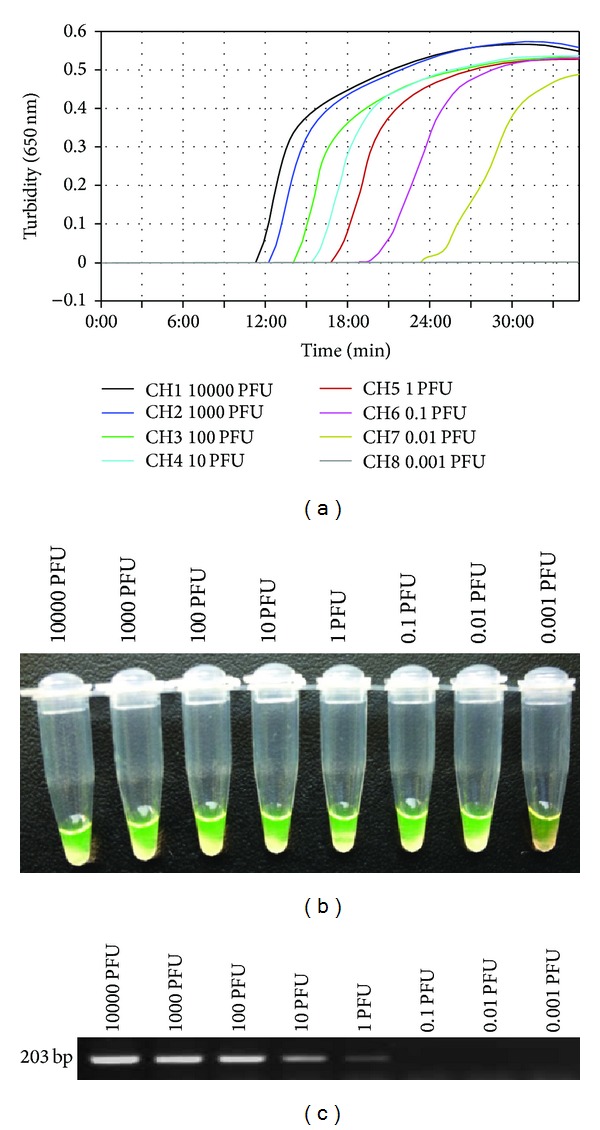
Comparative sensitivity of the RT-LAMP and RT-PCR methods. N9-RT LAMP and RT-PCR were performed using A/Shanghai/4664T/2013 (H7N9) viral RNA at concentrations ranging from 10,000 to 0.001 PFU per tube. ((a) and (b)) Detection limit of N9-RT-LAMP. LAMP products were detected using a real-time turbidity assay with an LA-320c (a) and a fluorescence assay (b). (c) Detection limit for the one-step RT-PCR using the same RNA extracts that were used for N9-RT LAMP. The PCR products were observed in a 1.5% agarose gel that was stained with ethidium bromide.

To evaluate the sensitivity of the N9-RT-LAMP assay with the NA primer sets, the detection limit of the assay was determined by testing against 10-fold serial dilutions of the H7N9 influenza virus (A/chicken/Shanghai/S1053/2013(H7N9)), which has a defined plaque-forming unit (PFU) dose. The kinetic analysis of the turbidity revealed that the N9-RT-LAMP assay was able to detect the H7N9 influenza virus at a level of 0.01 PFU per tube in less than 30 min (Figure 3(a)). N9-RT-LAMP sensitivity was also confirmed by observing the solution fluorescence under a UV light source. As shown in Figure 3(b), clear fluorescence signals were observed at concentrations ranging from 1000 to 0.01 PFU per tube. There were no differences in sensitivity between the real-time turbidity and visual fluorescence detections that were associated with the LAMP assay. When the same RNA template was subjected to one-step RT-PCR using the N9-specific primers, the detection limit of the system was 1 PFU per tube (Figure 3(c)). The results indicate that the sensitivity of N9-RT-LAMP assay is approximately 100-fold higher than that of RT-PCR.

The detection limits of H7N9 influenza viruses that were isolated from various sources were determined to assess whether the N9-RT-LAMP assay is able to detect a variety of H7N9 influenza viruses at constant sensitivity. The sensitivity, which was determined by the real-time monitoring of the turbidity of H7N9 viruses that were isolated from chicken, A/chicken/Shanghai/S1053/2013 (H7N9), from pigeon, A/pigeon/Shanghai/S1069/2013 (H7N9), from human, A/Anhui/1/2013 (H7N9), and from the environment, A/environment/Shanghai/S1088/2013 (H7N9), was 0.01 PFU per tube (Figures 2(e)–2(h)). Thus, N9-RT-LAMP was adapted for the detection of H7N9 viruses that were isolated from various sources.

### 3.4. RT-LAMP Specificity

**Figure 4:**
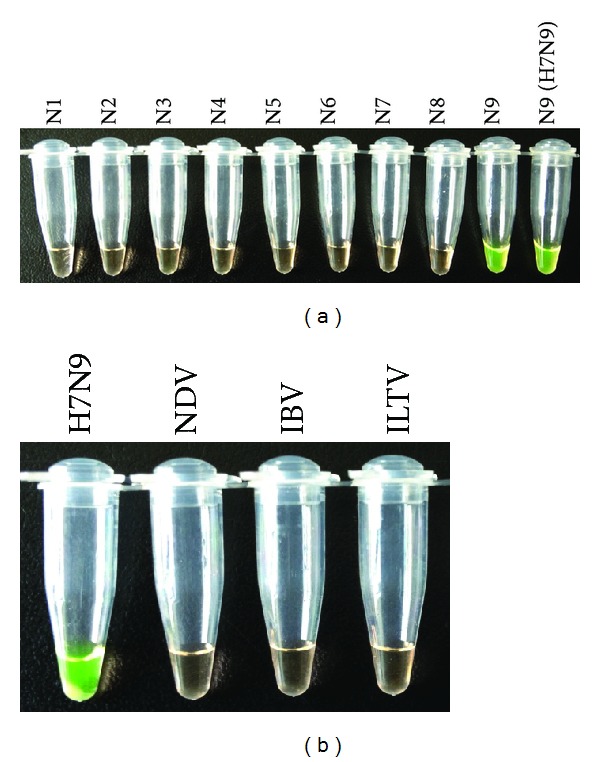
Specificity of the N9-RT-LAMP assay for different subtype influenza viruses and other avian respiratory viruses. Viral RNAs were extracted from the representative influenza viruses of avian origin (subtypes N1–9) (a) and other avian respiratory viruses (b). The RT-LAMP reaction was assessed based on the fluorescence intensity. N1, A/Goose/Guangdong/1/96 (H5N1); N2, A/Turkey/Wisconsin/1/66 (H9N2); N3, A/Duck/Germany/1215/73 (H2N3); N4, A/Turkey/Ontario/6118/68 (H8N4); N5, A/Mallard/Gurjev/263/82 (H14N5); N6, A/Duck/Czech/56 (H4N6); N7, A/equine/jingfang/1/74 (H7N7); N8, A/Duck/Ukraine/1/63 (H3N8); N9, A/Duck/Memphis/546/76 (H11N9); N9 (H7N9), A/chicken/Shanghai/S1053/2013;.

The N1–N9 subtype influenza viruses and three other avian respiratory viruses (NDV, IBV, and ILTV) were tested using the N9-RT-LAMP assay. Influenza virus (A/chicken/Shanghai/S1053/2013(H7N9)) was used as the positive control, and reactions that were performed in the absence of the template were used as negative controls. Only the H7N9 influenza virus was positive, and no LAMP products were detected in the reactions that were performed with RNA that was harvested from avian influenza viruses of different subtypes (Figure 4(a)) or from RNAs that were collected from other avian respiratory viruses (Figure 4(b)). These results demonstrated that the N9-RT-LAMP assay was specific and could be used to specifically detect the N9 subtype influenza virus.

### 3.5. Evaluation of the N9-RT-LAMP Assay Using Clinical Samples

To evaluate the ability of the N9-RT-LAMP assay to detect H7N9 viruses from clinical samples, 259 clinical samples were collected from three live poultry markets in Shanghai. All samples were tested by RT-LAMP and RT-PCR. Of the 259 samples that were evaluated, N9-RT-LAMP gave 18 positive cases, whereas RT-PCR gave 16 positive cases (Table 3). The positive rates after RT-LAMP and RT-PCR were 6.9% (18/259) and 6.2% (16/259), respectively. H7N9 viruses were isolated from 18 of the 18 positive samples by RT-LAMP. The results indicated that two N9-RT-LAMP-positive specimens were missed by RT-PCR.

## 4. Discussion

The novel influenza H7N9 virus has emerged and raises serious concerns for public health [1, 17]. No vaccine for the prevention of avian influenza A (H7N9) infections in humans and animals is currently available [18]. At present, the control of H7N9 virus infections primarily depends on the early identification and the quarantine of infected cases to prevent the further spread of the virus. Therefore, the development of a simple and rapid diagnostic method for this H7N9 virus is extremely important. In this study, specific, sensitive, and quick RT-LAMP methods were developed for H7N9 virus detection. RT-LAMP methods can detect different sources of the H7N9 virus (isolated from humans, chickens, pigeons, and the environment) in 30 min.

The H7-RT-LAMP assay was previously developed based on the HA genes in our lab [16]. We evaluated the ability of the H7-RT-LAMP assay to detect different sources of H7N9 viruses. The results indicated that the H7-RT-LAMP assay could detect as low as 0.01 PFU of different sources of H7N9 virus. This observation is consistent with the sensitivity of the assay for the detection of LPAI-H7N2 [16]. We compared the H7-RT-LAMP primer sequences and target sequences of the H7N9 virus. There are two nucleotide mismatches in the H7-RT-LAMP primer between H7N9 influenza viruses. The results of sensitivity indicated that the two nucleotide mismatches did not influence the amplification efficiency of the H7-RT-LAMP primer for the detection of the H7N9 virus.

We investigated the utility of NA-specific RT-LAMP primer sets for the rapid and accurate detection of different sources of H7N9 influenza virus RNA (from chickens, pigeons, the environment, and humans). The N9-RT-LAMP assay detected different sources of H7N9 influenza virus with a detection limit of 0.01 PFU (Figures 2 and 3). When the sensitivity of N9-RT-LAMP was compared with that of one-step RT-PCR using an H7N9 influenza virus dilution series, N9-RT-LAMP was approximately 100-fold more sensitive than RT-PCR. Two clinical samples tested negative by the RT-PCR but tested positive by the N9-RT-LAMP assay and by virus isolation. The results further indicated that the H7N9 virus RT-LAMP assay was slightly more sensitive than the RT-PCR for the detection of the H7N9 virus NA gene in specimens.

In addition, there is no cross-reactivity with other NA subtype influenza virus RNAs or other avian respiratory virus RNAs, suggesting that H7N9 virus NA-specific RT-LAMP has high specificity among some common avian viruses at the RNA level.

Since April 2013, H7N9 viruses that are similar to those viruses that were isolated from patients have been isolated from pigeons and chickens [1, 3]. The viruses can efficiently transmit among poultry, particularly chickens, and have spread from live poultry markets in Shanghai to eight other provinces in a relatively short period [4, 19, 20]. The viruses are nonpathogenic to poultry, which enable the avian H7N9 virus to replicate silently in avian species, and are severe threat to human health [4]. Thus, the timely surveillance of samples that are involved in poultry is extremely important for monitoring the prevalence of the H7N9 virus [21–23]. To evaluate the practicability of H7N9 virus RT-LAMP assays for the detection of avian-origin samples in this study, many clinical samples (including drinking water, soil, cloacal swab, and tracheal swab samples) that were collected from live poultry markets, poultry farms, and wild bird habitats from Shanghai and Anhui were tested by the H7N9 virus RT-LAMP assays. The results of the H7N9 virus RT-LAMP assays were consistent with those of the virus isolation [3]. Although 30 min was used for the H7N9 RT-LAMP reactions, most of the amplification reactions for clinical samples could be finished within 25 min. These results suggested that the H7N9 virus RT-LAMP assays were efficient, practical, and rapid diagnostic methods for the detection of the influenza A (H7N9) virus from different sources.

In summary, H7N9 virus RT-LAMP assays were developed and validated in this study for the detection of the H7N9 influenza virus with high sensitivity and specificity. The RT-LAMP assays required minimal laboratory equipment and could detect different sources of H7N9 influenza viruses. Moreover, compared with the requirements that are associated with RT-PCR and with virus isolation procedures, H7N9 virus RT-LAMP assays were significantly more rapid. Obtaining results within 2 h (including the extraction of RNA) is important for the rapid detection of the H7N9 influenza virus infection; thus, the present methods may be useful for the rapid detection of humans or animals that have been infected by the H7N9 influenza virus. Although FAO recommended that the diagnostic method for the H7N9 virus was the real-time RT-PCR (rRT-PCR) assay (http://www.who.int/influenza/gisrs_laboratory/cnic_realtime_rt_pcr_protocol_a_h7n9.pdf) [24], rRT-PCR requires an expensive machine system and experienced laboratory workers. We hope that this new, effective, and rapid diagnostic method will contribute to the control of the H7N9 influenza virus infection.

## 5. Conclusion

The specific, sensitive, and quick RT-LAMP methods were developed for H7N9 influenza virus detection. The RT-LAMP methods can detect the H7N9 virus from different resource samples (isolated from humans, chickens, pigeons, and the environment) in less than 30 min with high sensitivity and specificity. The RT-LAMP methods will contribute to the control of the H7N9 influenza virus infection.

## Figures and Tables

**Table 1:** Different subtypes of avian influenza viruses used for validation and specificity of RT-LAMP.

Subtype	Virus	Subtype	GenBank accession number
N1	A/Goose/Guangdong/1/96	H5N1	AF144305
N2	A/Turkey/Wisconsin/1/66	H9N2	D90305
N3	A/Duck/Germany/1215/73	H2N3	AY207522
N4	A/Turkey/Ontario/6118/68	H8N4	D90305
N5	A/Mallard/Gurjev/263/82	H14N5	M35997
N6	A/Duck/Czech/56	H4N6	D90306
N7	A/equine/jingfang/1/74	H7N7	—
N8	A/Duck/Ukraine/1/63	H3N8	V01087
N9	A/chicken/Shanghai/S1053/2013	H7N9	CY146958

**Table 2:** Details of RT-LAMP and RT-PCR primers designed for detection of NA gene sequences of N9 subtype influenza viruses.

Primer name^a^	Genome position	Length(s)	Sequences (5′ to 3′)
N9-F3		18-mer	GTTTCATGCGACCCAGAT
N9-B3		20-mer	CATGGCAACTAGTACTTGAC
N9-FIP		41-mer	TTCCGTTTGAGTGTTTCCCTCGAATGCAGGTTCTATGCTCT
N9-BIP		39-mer	CAGTATCGCGCCCTGATAAGCCACCCAATGCATTCCACC
N9-LB		23-mer	TGGCCACTATCATCACCGCCCAC
N9-LF		18-mer	TGATTGTTGTTCCTTGGC
N9 F		22-mer	ATAATGAAACAAACATCACCAA
N9 R		22-mer	AGCATAGAACCTGCATTCATCT

^a^The primers of N9-F3, N9-B3, N9-FIP, N9-BIP, N9-LB, and N9-LF were for RT-LAMP Primers. N9-F and N9-R were applied to RT-PCR.

**Table 3:** Detection of H7N9 influenza viruses in clinical samples by virus isolation, RT-LAMP, and RT-PCR.

Source of samples^a^	Total number of samples	H7N9 virus positive samples		
		Virus isolation	RT-LAMP	RT-PCR
Chickens	142	10	10	10
Pigeons	33	3	3	3
Ducks	30	0	0	0
Environment^b^	54	5	5	3

Total	259	18	18	16

^a^All samples were collected from poultry farms and live poultry markets in Shanghai and Anhui Province.

^b^The samples collected from the environment included soil, water, and fecal samples from different areas of the markets.
